# Association of lipoprotein lipase (LPL) gene variants with hyperlipidemic acute pancreatitis in southeastern Chinese population

**DOI:** 10.20945/2359-4292-2023-0195

**Published:** 2024-03-21

**Authors:** Yingyi Li, Hehui Cai, Yancheng Lin, Zhipeng Huang, Apei Zhou, Tianhao Huang, Yue-e Zeng, Meizhen Ye, Guiyuan Guo, Zicheng Huang

**Affiliations:** 1 The First Hospital of Quanzhou Affiliated to Fujian Medical University Department of Gastroenterology Quanzhou China Department of Gastroenterology, The First Hospital of Quanzhou Affiliated to Fujian Medical University, Quanzhou, People's Republic of China; 2 The First Hospital of Quanzhou Affiliated to Fujian Medical University Clinical Laboratory Quanzhou People's Republic of China Clinical Laboratory, The First Hospital of Quanzhou Affiliated to Fujian Medical University, Quanzhou, People's Republic of China; 3 HI. Q Biomedical Laboratory Quanzhou People's Republic of China HI. Q Biomedical Laboratory, Third Floor Building 5 Derun Industrial Park, Taiwan Investment Zone, Quanzhou, People's Republic of China

**Keywords:** LPL, rs371282890, hyperlipidemic acute pancreatitis, L279V

## Abstract

**Objective:**

The study aims to explore the relationship between lipoprotein lipase *(LPL)* variants and hyperlipidemic acute pancreatitis (HLAP) in the southeastern Chinese population. Subjects and methods: In total, 80 participants were involved in this study (54 patients with HLAP and 26 controls). All coding regions and intron-exon boundaries of the *LPL* gene were sequenced. The correlations between variants and phenotypes were also analysed.

**Results:**

The rate of rare *LPL* variants in the HLAP group is 14.81% (8 of 54), higher than in controls. Among the detected four variants (rs3735959, rs371282890, rs761886494 and rs761265900), the most common variant was rs371282890. Further analysis demonstrated that subjects with rs371282890 "GC" genotype had a 2.843-fold higher risk for HLAP (odds ratio [OR]: 2.843, 95% confidence interval [CI]: 1.119-7.225, *p* = 0.028) than subjects with the "CC" genotype. After adjusting for sex, the association remained significant (adjusted OR: 3.083, 95% CI: 1.208-7.869, p = 0.018). Subjects with rs371282890 "GC" genotype also exhibited significantly elevated total cholesterol, triglyceride and non-high-density lipoprotein cholesterol levels in all the participants and the HLAP group (*p* < 0.05).

**Conclusion:**

Detecting rare variants in *LPL* might be valuable for identifying higher-risk patients with HLAP and guiding future individualised therapeutic strategies.

## INTRODUCTION

Hyperlipidemic acute pancreatitis (HLAP), which refers to acute pancreatitis (AP) due to hypertriglyceridemia (HTG) (1), is the second leading cause of AP in China (2). With the continuous improvement of socioeconomic levels and people's living standards, the prevalence of dyslipidemia (3) and HLAP (2,4,5) trends has increased yearly. Recent studies have shown that the risk of AP increases with the rise in serum triglyceride (TG) levels. When the TG level ≥ 5.65 mmol/L, the risk of AP increases significantly (6). The incidence of AP reaches up to 5% and 10%~20% in patients with serum TG levels >11.3 mmol/L and > 22.6 mmol/L, respectively (7). HLAP is more likely to get aggravated than other AP causes (8, 9). In addition, higher TG levels independently correlate to an elevated rate of complications and a more severe course of disease (10). Timely reduction of serum TG levels should be the key to halting the disease's progression and reducing mortality. Moreover, HLAP presents a higher incidence of relapse (10). About 32% of the patients with HLAP have had a recurrence in a large cohort study of HTG and patients with pancreatitis (11). Maintaining normal blood lipid levels is a practical approach to prevent AP recurrence.

The aetiology of HLAP is complicated and could be caused by interactions between genetic and environmental factors. Primary HTG, also known as familial chylomicronemia (FCS), occurs when there are homozygous or compound heterozygous rare variants in six canonical genes involved in the TG metabolism, such as lipoprotein lipase (*LPL*), apolipoprotein C2 (*APOC2*), GPI-anchored HDL-binding protein 1 (*GPIHBP1*), apolipoprotein A5 (*APOA5*), lipase maturation factor 1 (*LMF1*) and glucokinase regulator protein (*GCKR*) (12,13). Recurrent pancreatitis is a clinical manifestation of the disease. However, multifactorial chylomicronemia syndrome (MCS) is much more clinically prevalent than FCS. People with a heterozygous rare variant or several common small-effect single-nucleotide polymorphisms (SNPs) in one of the six canonical genes involved in the TG metabolism are predisposed to MCS (12). It occurs due to genetic susceptibility and secondary factors, such as a high-fat diet, obesity, uncontrolled diabetes and alcohol intake. Patients with MCS also face a high risk of recurrent pancreatitis (12). Previous studies demonstrated genetic features of hypertriglyceridemia or chylomicronemia in China (14-16). However, the genetic factor of HLAP is not yet adequately studied. LPL is the crucial enzyme of lipid metabolism that degrades triglycerides. Recently, some rare variants in the *LPL* gene were reported in type 1 hyperlipoproteinemia and patients with HLAP (17-27). A study in Taiwan has demonstrated that the rate of mutations in the *LPL* gene reached 17% among 53 cases of patients with HLAP. In contrast, 77.8% of patients with HTG and variants of the *LPL* gene have HLAP (28). However, Xiao-Yao Li and cols. demonstrated that not all patients had homozygote *LPL* variants and that LPL deficiency would develop AP (22). Studies with larger sample sizes are needed to confirm the association of *LPL* variations in HLAP.

This study aims to investigate the genetic characteristics of patients with HLAP, determine the correlations between HLAP and the rare variants of the *LPL* gene, offer fresh insights into the complex aetiology of HLAP, and provide new targets and directions for the individualised treatment of patients with HLAP.

## SUBJECTS AND METHODS

### Subjects

In total, 80 participants were recruited from the Fujian Medical University Affiliated First Quanzhou Hospital in southeastern China from 2018 to 2022. This research enrolled 34 patients with HLAP as cases and 26 patients with biliary acute pancreatitis (BAP) as controls. The diagnostic criteria for HLAP were: (a) the diagnosis of AP according to the Chinese guidelines for the management of AP (fulfilling at least two of the following three features: upper abdominal pain consistent with AP, serum lipase activity exceeds the upper limit of normal by three times, and radiographic features of AP); (b) confirmed dyslipidemia with serum TG >11.3 mmol/L or serum TG >5.65 mmol/L together with lipemia. The diagnostic criteria for the control group were: (a) the diagnosis of AP according to Chinese recommendations for the management of AP; (b) confirmed gallstones or biliary sludge on imaging methods or elevated serum levels of alanine aminotransferase (ALT) (>60 U/L). The exclusion criteria included patients with a history of alcohol consumption of more than 20 g/day, consumption of pancreatic toxic drugs, known pancreatic or periampullary tumours, pancreatic duct anomaly disorders, hereditary pancreatitis, autoimmune pancreatitis and traumatic pancreatitis. The Ethics Committee of the Fujian Medical University Affiliated First Quanzhou Hospital approved this study's protocols.

### Information collection of general characteristics and biochemical measurements

The general clinical information, such as age, gender, alcohol consumption, smoking status, age of first onset, hospitalisation frequency and total hospitalisation cost, was collected via medical history examination and a questionnaire. Blood samples were collected in the morning after an overnight fast to detect biochemical parameters that include lipid and glycaemic profiles.

### Genotyping analysis

The genomic DNA was extracted from peripheral blood lymphocytes. PCR reactions were performed on gDNA with each primer pair of the target gene. The PCR products of the expected size were separated by 1% agarose gel electrophoresis to confirm the presence and specificity of targeted sequences. Following further purification, the PCR products were sequenced by the dideoxy approach. DNA Baser v5.15.0 aligned the DNA fragments to target the references and detect the mutations from chromatograms.

### Statistical analysis

Quantitative variables were expressed as mean ± standard deviation. Differences in the characteristics between groups were examined by the Student's *t*-test or Wilcoxon test. The chi-square test was applied to evaluate the categorical variables between groups. The Cox regression was used to calculate the HR value of the genotypes. Moreover, the Cox ratio risk regression model was applied to analyse the effects of genotype and gender on HLAP events. Statistical analysis was performed using SPSS 22.0 software, and two-tailed (*p* < 0.05) was considered statistically significant in this study.

## RESULTS

### General characteristics

Table 1 presents the general characteristics of the 54 patients with HLAP and the 26 controls with BAP. The mean age in the HLAP group was younger. The proportion of males in the HLAP group was more than that of females (*p* < 0.05). The mean total cholesterol (TC), TG, non-high-density lipoprotein cholesterol (non-HDL-C) and glycosylated haemoglobin (HBA) were significantly higher in the HLAP group than in the controls (*p* < 0.05). The proportion of smokers, drinkers and patients with fatty liver and diabetes was higher in the HLAP group (*P* < 0.05). More patients with HLAP had a relapse than controls (*p* < 0.05).

**Table 1:** Clinical and biochemical characteristics of patients with HLAP and controls

	Patients with HLAP (*n* = 54)	BAP controls (*n* = 26)	t (*χ*^2^)	p-value
Male, n (%)	39 (72.2%)	11 (42.3%)	6.701	0.010
Age (years)	38.80 ± 10.19	58.04 ± 14.04	–6.972	<0.001
SBP (mm Hg)	133.98 ± 16.25	140.12 ± 27.07	–1.067	0.294
DBP (mm Hg)	85.39 ± 11.51	82.96 ± 16.82	0.665	0.510
TG (mmol/L)	28.38 ± 18.99	1.30 ± 0.66	10.465	<0.001
TC (mmol/L)	11.57 ± 4.26	4.61 ± 0.91	11.493	<0.001
HDL-C (mmol/L)	0.89 ± 0.76	1.08 ± 0.32	–1.201	0.233
LDL-C (mmol/L)	2.29 ± 1.93	2.96 ± 0.63	–2.256	0.027
Non-HDL-C (mmol/L)	10.71 ± 4.03	3.53 ± 0.79	12.390	<0.001
FPG (mmol/L)	11.39 ± 6.08	11.88 ± 5.02	1.641	0.105
HBA (%)	8.59 ± 2.79	6.24 ± 1.18	5.190	<0.001
Drinking, *n* (%)	22 (40.7%)	2 (7.7%)	9.128	0.003
Smoking, *n* (%)	23 (42.6%)	0(0.0%)	15.543	<0.001
Hypertension, *n* (%)	10 (18.5%)	10 (38.5%)	3.723	0.054
Fatty liver, *n* (%)	47 (87.0%)	11 (42.3%)	17.611	<0.001
Diabetes, *n* (%)	33 (61.1%)	6 (23.1%)	10.161	0.001
Cholelithiasis, *n* (%)	5 (9.3%)	25 (96.2%)	56.539	<0.001
Hypertriglyceridemia, *n* (%)	54 (100.0%)	6 (23.1%)	55.385	<0.001
MSAP and SAP, *n* (%)	37 (68.6%)	18 (68.8%)	0.678	0.713
Recurrent AP, *n* (%)	30 (55.6%)	8 (30.8%)	4.324	0.038

Results are expressed as mean ± SD. SBP: systolic blood pressure; DBP: diastolic blood pressure; TC: total cholesterol; TG: triglycerides; HDL-C: high-density lipoprotein cholesterol; LDL-C: low-density lipoprotein cholesterol; non-HDL-C: non-high-density lipoprotein cholesterol; FPG: fasting plasma glucose; HBA: glycated haemoglobin; MSAP: moderately severe acute pancreatitis; SAP: severe acute pancreatitis; P < 0.05 was considered statistically significant.

### LPL gene variants

The rate of *LPL* gene mutation in the HLAP group is 14.81% (8 of 54). Table 2 shows the *LPL* variants detected in the HLAP group and controls, including four rare variants (rs3735959, rs371282890, rs761886494 and rs761265900). All the mutations were present in the heterozygous state, except one patient carried a compound heterozygous mutation of rs371282890 and rs761886494, which is reported as pathogenic or likely pathogenic in ClinVar.

**Table 2:** The distribution of *LPL* gene variants detected in patients with HLAP and controls

SNP	Minor allele	N, MAF		*χ* ^2^	*p-*value	Clinical significance reported in ClinVar
		Patients with HLAP (*n* = 54)	BAP controls (*n* = 26)			
rs3735959	C	2 (0.020)	2 (0.042)	0.546	0.460	Benign/Likely benign
rs371282890	G	5 (0.046)	0 (0)	2.485	0.175	Pathogenic
rs761886494	A	1 (0.009)	0 (0)	0.498	1.000	Likely pathogenic
rs761265900	G	1 (0.009)	0 (0)	0.498	1.000	Not reported

SNP: single-nucleotide polymorphism; MAF: minor allele frequency; p-value < 0.05 was considered statistically significant.

### LPL rs371282890 and the risk of HLAP

**Figure 1:**
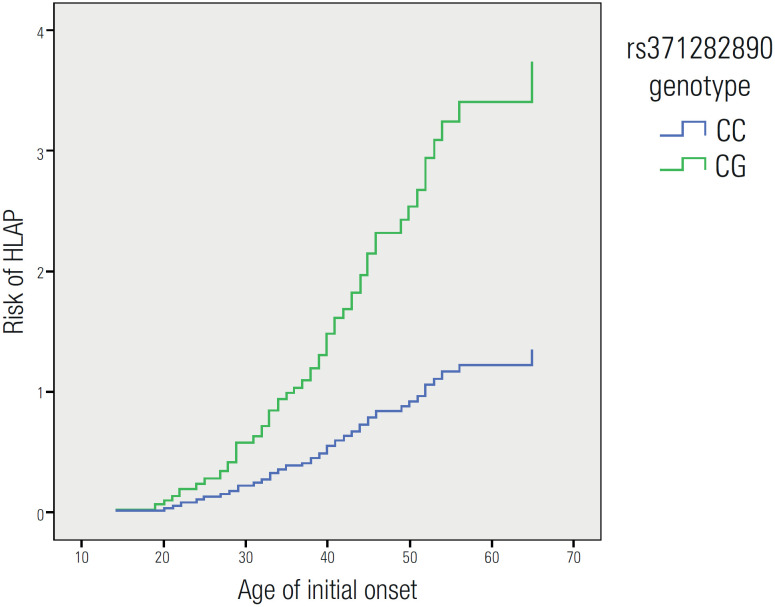
Different *LPL* rs371282890 genotypes and risk of HLAP.

Figure 1 depicts that different *LPL* rs371282890 genotypes have distinct risks towards HLAP. Subjects with the "GC" genotype had a 2.843-fold higher chance of HLAP (HR: 2.843, 95% confidence interval [CI]: 1.119-7.225, *p* = 0.028) than subjects with the "CC" genotype. After controlling for sex, patients with the "GC" genotype also had a 3.083-fold higher risk for HLAP (HR: 3.083, 95% CI: 1.208-7.869, *p* = 0.018) than subjects with the "CC" genotype (Table 3).

**Table 3:** Cox regression analysis of the impact of gender and rs371282890 on HLAP

Factor		Age of first onset (years)	β	p-value	HR	95%CI
Gender						
	Male	39.24	0.937	0.002	2.551	1.398-4.655
	Female	50.87	–	–	1.000	-
rs371282890 genotype						
	GC	32.20	1.126	0.018	3.083	1.208-7.869
	CC	44.36	–	–	1.000	–

p-value <0.05 was considered statistically significant.

### Clinical characteristics and metabolic indexes between different *LPL* rs371282890 genotypes

Table 4 presents the clinical characteristics, lipid parameters and glycemia parameters between *LPL* rs371282890 "GC" and "CC" genotypes. Compared with the "CC" genotype, subjects with genotype "GC" had significantly increased TC, TG and non-HDL-C levels in all participants and in the HLAP group (*p* <0.05). The mean age, hospitalisation frequency, HDL-C, LDL-C, FPG and HBA levels did not differ between the two genotypes.

**Table 4:** Differences of characteristics and parameters between rs371282890 genotypes (GC, CC)

rs371282890	Genotype		
	GC (*n* = 5)	CC in HLAP group (*n* = 49)	CC in all subjects (*n* = 75)
Age (year)	34.60 ± 10.71	39.22 ± 10.15	45.75 ± 14.65
TC (mmol/L)	15.99 ± 5.15	11.12 ± 3.95+	8.86 ± 4.49*
TG (mmol/L)	53.82 ± 22.07	17.29 ± 17.93+	17.29 ± 17.93*
HDL-C (mmol/L)	1.95 ± 1.75	0.78 ± 0.50	0.89 ± 0.46
LDL-C (mmol/L)	2.05 ± 2.99	2.32 ± 1.83	2.55 ± 1.54
Non-HDL-C (mmol/L)	14.04 ± 3.87	10.36 ± 3.92+	7.92 ± 4.57*
FPG (mmol/L)	11.88 ± 5.02	12.20 ± 6.42	11.39 ± 6.08
HBA (%)	9.14 ± 2.91	8.53 ± 2.80	7.76 ± 2.61

Results are expressed as mean ± SD. TC: total cholesterol; TG: triglycerides; HDL-C: high-density lipoprotein cholesterol; LDL-C: low-density lipoprotein cholesterol; Non-HDL-C: non-high-density lipoprotein cholesterol; FPG: fasting plasma glucose; HBA: glycated haemoglobin; p-value < 0.05 was considered statistically significant.

+ p < 0.05 for statically significant difference between the GC group and the CC in the HLAP group.

* p < 0.05 for statically significant difference between the GC group and the CC in all subjects.

## DISCUSSION

HLAP is a very heterogeneous disease. Many patients with HLAP struggle to consistently achieve the threshold of TG levels required for preventing pancreatitis and are beset by the continued relapse of pancreatitis throughout their lifespan. Genetics could be a critical factor in the susceptibility to lipid metabolism abnormality and HLAP onset. However, whether and how genes involving lipid metabolism affect the occurrence of HLAP is not completely clear. This study compared the clinical characteristics and rare variants in the *LPL* gene of patients with HLAP and patients with BAP who have or do not have dyslipidemia. Several rare variants were observed in the HLAP group, especially rs371282890 (L279V). Furthermore, the lipid parameters of patients with and without variant rs371282890 were compared. The findings revealed that variant rs371282890 carriers have more pronounced lipid metabolism abnormalities.

LPL is the crucial enzyme of lipid metabolism, which hydrolyses circulating TGs packaged in chylomicrons or very low-density lipoproteins. The reduced LPL activity causes an increase in plasma TG levels, contributing to a higher risk of AP. A recent study revealed that patients with severe hypertriglyceridemia (STG) having a history of AP had a higher frequency of the rare variants in the *LPL* gene and all the LPL molecular regulating genes (14). The intensive genetic analysis for Chinese patients with TG ≥ 5.65 mmol/L exhibited 5 (27.8%) of 14 patients with AP had *LPL* variations (14). Khovidhunkit and cols. also reported that among 13 Thai patients with AP history, 3 (23.1%) had rare variants of *LPL* (29). In contrast, another study from China only found six rare variants in other LPL molecular regulating genes (*APOA5, GPIHBP1* and *LMF1*). No *LPL* variations were found in 11 patients with HLAP (30). So far, there is no comprehensive investigation of the genetic characteristics of HLAP in China. Therefore, this study added more information to this field. This study identified four rare variants (rs3735959, rs371282890, rs761886494 and rs761265900) in *LPL*. Moreover, 8 (14.8%) of 54 patients with HLAP had *LPL* variations. The difference in the frequency of rare mutations between this study and the previous studies may be attributed to ethnic differences and selection bias. Moreover, the higher frequency of *LPL* variations in patients with HLAP was visible but not statistically provable, which was similar to those previous studies.

Previous case reports and cases series have revealed that some *LPL* mutations are associated with HLAP in China, such as p.W14X, p.Gln188*, p.His210Leu, p.Glu242Lys, p.Leu252VaL, p.Cys264Ter and p.L279V (19,22,27,31,32). Among them, L279V in exon 6 of *LPL* was first reported to be associated with HLAP from the Chinese population. Chen and cols. discovered a compound heterozygote for a missense mutation A98T and a missense mutation L279V in two probands with SHTG and AP. And beyond that, one family member and one out of 70 other HTG subjects also carried this novel *LPL* L279V mutation (33). Li and cols. also reported a compound heterozygous for W14X and L279V *LPL* gene mutations in a Chinese patient with long-term severe hypertriglyceridemia and recurrent AP (32). Khovidhunkit and cols. also observed that one in three patients with HLAP had rare variants in *LPL* carried L279V (29). In Jing-Lu Jin's study, the expanding genetic research included 15 TG-related genes in 103 patients with primary SHTG, and one patient carried L279V out of 46 patients with rare variants (14). Other previous studies and reports have seldom discovered this mutation. Distinguished from these previous studies, the most frequent mutation detected in this study was rs371282890 (L279V). Five patients carried the rs371282890 variant in the HLAP group, more than those in previous studies. *LPL* Exon 6 (residues 232-313) encodes two structurally relevant disulphide bridges (Cys278-Cys283 and Cys264-Cys275) for the binding of heparin (34). The mutation L279V constitutes one of these disulphide bridges (Cys278-Cys283), which is crucial for the catalytic function of heparin binding. The L279V site is conserved throughout evolution from chimpanzees to zebrafish, suggesting that this residue may play a critical role in LPL function (33). This study compared the risk of AP and lipid metabolism in different rs371282890 genotypes. These five patients with the rs371282890 "GC" genotype had a higher chance of HLAP (HR: 2.843, 95% CI: 1.119-7.225, *p* = 0.028). Moreover, these five patients also had higher TG, TC, and non-HDL-C levels in all participants and the HLAP group. But whether rs371282890 variants can confer the risk for HLAP by simply increasing the serum TG or through other complex mechanisms need further research.

Conventional pharmacological therapies lower TG predominantly by the stimulation of LPL activity and are less effective in the case of a severely dysfunctional LPL protein (35). Due to the costs of genetic analysis and a lack of attention to molecular diagnosis, this group of patients with molecular `defects’ fail to devise in-depth diagnostic strategies. A routine sequencing of the canonical genes involved in TG metabolism (*LPL, APOC2, GPIHBP1, APOA5, LMF1* and *GCKR*) in patients with HLAP may help identify this group and develop differential therapeutic strategies. Other than the strict low-fat diet, novel medications targeting TG-related processes, like inhibitors of APOC3, ANGPTL4, MGAT and DGAT, may reduce the risk of HLAP (35). The study has some limitations to be acknowledged. First, the study cohort was small in size. Hence, this study's subjects may not entirely represent the Chinese population. Second, LPL mass and activity measurements were not performed, thus hampering the pathogenetic interpretations of genotyping. Third, there was a difference in age between the two groups due to the features of the disease itself, so maybe some selection biases existed. Further studies are required to confirm the results. Another study limitation is that no other lipid metabolism genes were analysed. Expanded genetic testing in the future will generate more genetic information about HLAP.

In conclusion, detecting rare variants in *LPL* might help identify the portion of patients with HLAP at higher risk and guide future individualised therapeutic strategies. *LPL* variant rs371282890 is associated with HLAP and severe HTG in southeastern China.
